# Supplementary material for: Investigation of Sperm and Seminal Plasma Candidate MicroRNAs of Bulls with Differing Fertility and In Silico Prediction of miRNA-mRNA Interaction Network of Reproductive Function
Source: Animals (Basel). 2022 Sep 9;12(18):2360. doi: 10.3390/ani12182360 (PMC9495167; doi:10.3390/ani12182360)
Supplement: Supplementary file 1 [file animals-12-02360-s001.zip › Figure S1.pdf]

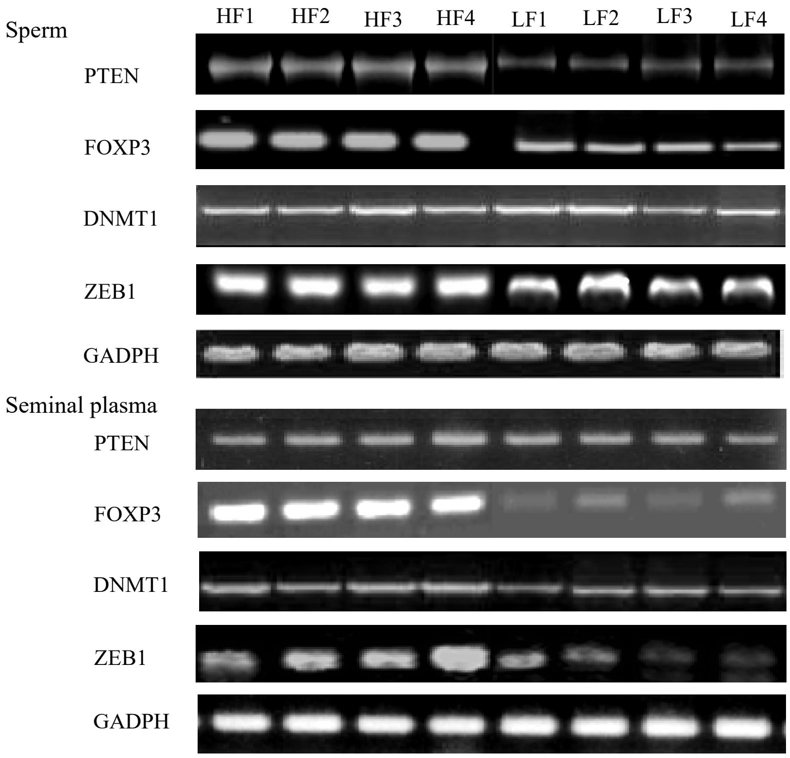

Figure S1. The ethidium bromide-stained electrophoresis gel, with amplicons of expected sizes.

Expected amplicons for each gene target, in sperm and seminal plasma for from four high fertile and four low fertile bulls, were shown.

HF, high fertile bull;
LF, low fertile bull;
DNMT1, dna methyltransferase 1;
FOXP3, forkhead box P3 (scurfin);
PTEN, phosphatase and tensin homolog;
ZEB1, Zinc Finger E-Box Binding Homeobox 1;
GADPH, glyceraldehyde-3-phosphate dehydrogenase;
