# Supplementary material for: Investigation of Sperm and Seminal Plasma Candidate MicroRNAs of Bulls with Differing Fertility and In Silico Prediction of miRNA-mRNA Interaction Network of Reproductive Function
Source: Animals (Basel). 2022 Sep 9;12(18):2360. doi: 10.3390/ani12182360 (PMC9495167; doi:10.3390/ani12182360)
Supplement: Supplementary file 1 [file animals-12-02360-s001.zip › Figure S2.pdf]

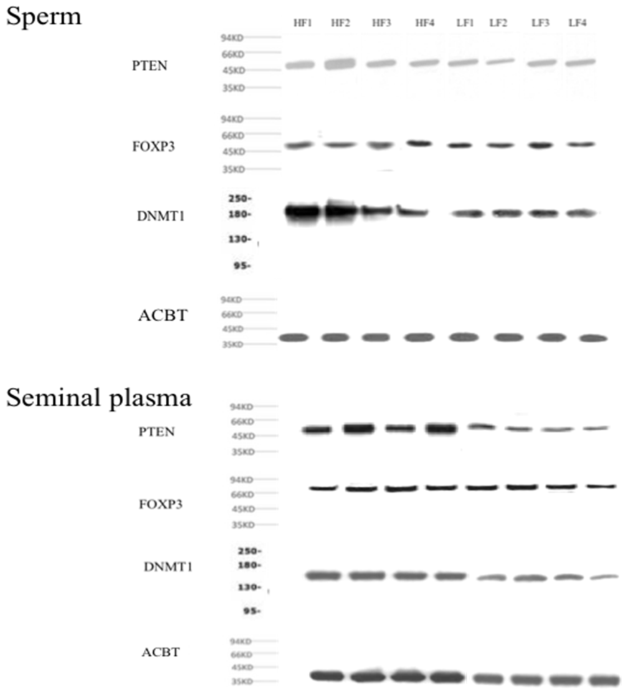

Figure S2. Representative Western blots of protein isozymes.

PTEN, phosphatase and tensin homolog, 47 kDa;
FOXP3, forkhead box P3 (scurfin), 47 kDa;
DNMT1, dna methyltransferase 1, ~180 kDa
ACTB, beta actin, 42 kDa;
