# Supplementary material for: Investigation of Sperm and Seminal Plasma Candidate MicroRNAs of Bulls with Differing Fertility and In Silico Prediction of miRNA-mRNA Interaction Network of Reproductive Function
Source: Animals (Basel). 2022 Sep 9;12(18):2360. doi: 10.3390/ani12182360 (PMC9495167; doi:10.3390/ani12182360)
Supplement: Supplementary file 1 [file animals-12-02360-s001.zip › Table S1.pdf]

Supplementary Table S1. Sire conception rate (SCR)*, progressive motility (%) and abnormal spermatozoa (%) for Holstein bulls used in the study

Bull	SCR	Breeding number	Reliability (%)	Progressive motility (%)	Abnormal spermatozoa (%)
HF1	+4.2	31609	91	90	12
HF2	+4.1	38493	90	90	8
HF3	+4.4	23912	90	85	10
HF4	+4.0	21453	92	90	12
LF1	-2.2	1442	81	85	10
LF2	-2.2	1448	80	80	13
LF3	-2.4	1536	84	85	16
LF4	-2.1	1491	83	90	12

*SCR is an indicator of the semen fertility of bulls used for artificial insemination and is the only national fertility evaluation in the US. The SCR evaluation model uses data from all four US Dairy Record Processing Centers, including data from all 50 states plus Puerto Rico and Mexico. SCR is expressed as a relative conception rate (CR) and is reported as a percentage to the nearest .1 %. SCRs are reported on a within breed basis. The average CR of all bulls has an average SCR of 0.0 %. A bull with an SCR of +4.0 % is expected to have a 4.0 % higher CR than an average bull and a 6.0 % higher CR than a bull with an SCR of -2.0 %. A bull with an SCR of +2.0 % is expected to have a CR of 32 % in a herd that normally averages 30 % and historically has used bulls with average SCR.
