# Supplementary material for: Investigation of Sperm and Seminal Plasma Candidate MicroRNAs of Bulls with Differing Fertility and In Silico Prediction of miRNA-mRNA Interaction Network of Reproductive Function
Source: Animals (Basel). 2022 Sep 9;12(18):2360. doi: 10.3390/ani12182360 (PMC9495167; doi:10.3390/ani12182360)
Supplement: Supplementary file 1 [file animals-12-02360-s001.zip › Table S2.pdf]

Table S2. Nucleotide sequences of human, mouse, and cattle miRs (www.mirbase.org)

Upregulated miRNA	Sequence
has-miR-20b-5p<br>mmu-miR-20b-5p<br>bta-miR-20b	CAAAGUGCUCAUAGUGCAGGUAG<br>CAAAGUGCUCAUAGUGCAGGUAG<br>CAAAGUGCUCACAGUGCAGGUA
has-miR-214-3p<br>mmu-miR-214-3p<br>bta-miR-214	ACAGCAGGCACAGACAGGCAGU<br>ACAGCAGGCACAGACAGGCAGU<br>ACAGCAGGCACAGACAGGCAGU
hsa-miR-21-3p<br>mmu-miR-21-3p<br>bta-miR-21-3p	CAACACCAGUCGAUGGGCUGU<br>CAACAGCAGUCGAUGGGCUGUC<br>AACAGCAGUCGAUGGGCUGUCU
hsa-miR-193a-3p<br>mmu-miR-193a-3p<br>bta-miR-193a-3p	AACUGGCCUACAAAGUCCCAGU<br>AACUGGCCUACAAAGUCCCAGU<br>AACUGGCCUACAAAGUCCCAGU
hsa-miR-142-5p<br>mmu-miR-142-5p<br>bta-miR-142-5p	CAUAAAGUAGAAAGCACUACU<br>CAUAAAGUAGAAAGCACUACU<br>CAUAAAGUAGAAAGCACUAC
hsa-miR-199a-5p<br>mmu-miR-199a-5p<br>bta-miR-199a-5p	CCCAGUGUUCAGACUACCUGUUC<br>CCCAGUGUUCAGACUACCUGUUC<br>CCCAGUGUUCAGACUACCUGUU
hsa-miR-145-5p<br>mmu-miR-145-5p<br>bta-miR-145	GUCCAGUUUUCCCAGGAAUCCCU<br>GUCCAGUUUUCCCAGGAAUCCCU<br>GUCCAGUUUUCCCAGGAAUCCCU
hsa-miR-151-5p<br>mmu-miR-151-5p<br>bta-miR-151-5p	UCGAGGAGCUCACAGUCUAGU<br>UCGAGGAGCUCACAGUCUAGU<br>UCGAGGAGCUCACAGUCUAGU
hsa-miR-199a- 3p<br>mmu- miR-199a- 3p<br>bta- miR-199a- 3p	ACAGUAGUCUGCACAUUGGUUA<br>ACAGUAGUCUGCACAUUGGUUA<br>ACAGUAGUCUGCACAUUGGUUA
hsa-miR-31-5p<br>mmu-miR-31-5p<br>bta-miR-31	AGGCAAGAUGCUGGCAUAGCU<br>AGGCAAGAUGCUGGCAUAGCUG<br>AGGCAAGAUGCUGGCAUAGCU
hsa-miR-320a-3p<br>mmu-miR-320a-3p<br>bta-miR-320a	AAAAGCUGGGUUGAGAGGGCGA<br>---<br>AAAAGCUGGGUUGAGAGGGCGA
hsa-miR-484<br>mmu-miR-484<br>bta-miR-484	UCAGGCUCAGUCCCCUCCCGAU<br>UCAGGCUCAGUCCCCUCCCGAU<br>UCAGGCUCAGUCCCCUCCCGAU
hsa-miR-138-5p<br>mmu-miR-138-5p<br>bta-miR-138	AGCUGGUGUUGUGAAUCAGGCCG<br>AGCUGGUGUUGUGAAUCAGGCCG<br>AGCUGGUGUUGUGAAUCAGGCCG
hsa-miR-139-5p<br>mmu-miR-139-5p<br>bta-miR-139	UCUACAGUGCACGUGUCUCCAGU<br>UCUACAGUGCACGUGUCUCCAG<br>UCUACAGUGCACGUGUCUCCAGU
hsa-miR-150-5p<br>mmu-miR-150-5p	UCUCCCAACCCUUGUACCAGUG<br>UCUCCCAACCCUUGUACCAGUG

bta-miR-150	UCUCCCAACCCUUGUACCAGUGU
hsa-miR-107<br>mmu-miR-107-3p<br>bta-miR-107	AGCAGCAUUGUACAGGGCUAUCA<br>AGCAGCAUUGUACAGGGCUAUCA<br>AGCAGCAUUGUACAGGGCUAUC
hsa-miR-17-5p<br>mmu-miR-17-5p<br>bta-miR-17-5p	CAAAGUGCUUACAGUGCAGGUAG<br>CAAAGUGCUUACAGUGCAGGUAG<br>CAAAGUGCUUACAGUGCAGGUAGU
hsa-miR-193a-5p<br>mmu-miR-193a-5p<br>bta-miR-193a-5p	UGGGUCUUUGCGGGCGAGAUGA<br>UGGGUCUUUGCGGGCAAGAUGA<br>UGGGUCUUUGCGGGCGAGAUGA
hsa-miR-215-5p<br>mmu-miR-215-5p<br>bta-miR-215	AUGACCUAUGAAUUGACAGAC<br>AUGACCUAUGAUUUGACAGAC<br>AUGACCUAUGAAUUGACAGACA
**Downregulated miRNA**	**Sequence**
hsa-miR-16b<br>mmu-miR-16b<br>bta-miR-16b	--<br>--<br>UAGCAGCACGUAAAUAUUGGC
hsa-miR-29c-3p<br>mmu-miR-29c-3p<br>bta-miR-29c	UAGCACCAUUUGAAAUCGGUUA<br>UAGCACCAUUUGAAAUCGGUUA<br>UAGCACCAUUUGAAAUCGGUUA
hsa-miR-200a-3p<br>mmu-miR-200a-3p<br>bta-miR-200a	UAACACUGUCUGGUAACGAUGU<br>UAACACUGUCUGGUAACGAUGU<br>UAACACUGUCUGGUAACGAUGUU
hsa-miR-101a-3p<br>mmu-miR-101-3p<br>bta-miR-101	UACAGUACUGUGAUAACUGAA<br>UACAGUACUGUGAUAACUGAA<br>UACAGUACUGUGAUAACUGAA
hsa-miR-29a-3p<br>mmu-miR-29a<br>bta-miR-29a	UAGCACCAUCUGAAAUCGGUUA<br>UAGCACCAUCUGAAAUCGGUUA<br>CUAGCACCAUCUGAAAUCGGUUA
hsa-miR-34b-5p<br>mmu-miR-34b-3p<br>bta-miR-34b	UAGGCAGUGUCAUUAGCUGAUUG<br>AGGCAGUGUAAUUAGCUGAUUGU<br>AGGCAGUGUAAUUAGCUGAUUG
hsa-miR-186-5p<br>mmu-miR-186-5p<br>bta-miR-186	CAAAGAAUUCUCCUUUUGGGCU<br>CAAAGAAUUCUCCUUUUGGGCU<br>CAAAGAAUUCUCCUUUUGGGCU
hsa-miR-99a-5p<br>mmu-miR-99a-5p<br>bta-miR-99a-5p	AACCCGUAGAUCCGAUCUUGUG<br>AACCCGUAGAUCCGAUCUUGUG<br>AACCCGUAGAUCCGAUCUUGU
hsa-miR-15b-5p<br>mmu-miR-15b-5p<br>bta-miR-15b	UAGCAGCACAUCAUGGUUUACA<br>UAGCAGCACAUCAUGGUUUACA<br>UAGCAGCACAUCAUGGUUUACA
hsa-miR-128-3p<br>mmu-miR-128-3p<br>bta-miR-128	UCACAGUGAACCGGUCUCUUU<br>UCACAGUGAACCGGUCUCUUU<br>UCACAGUGAACCGGUCUCUUU
hsa-miR-148a-3p<br>mmu-miR-148a-3p	UCAGUGCACUACAGAACUUUGU<br>UCAGUGCACUACAGAACUUUGU

bta-miR-148a	UCAGUGCACUACAGAACUUUGU
hsa-miR-191-5p<br>mmu-miR-191-5p<br>bta-miR-191	CAACGGAAUCCCAAAAGCAGCUG<br>CAACGGAAUCCCAAAAGCAGCUG<br>CAACGGAAUCCCAAAAGCAGCUG

Note: Nucleotide sequences are conserved among human, mouse and cattle.
Yellow colored denote difference in sequences.
